# Type I–IV Halogen⋯Halogen Interactions: A Comparative Theoretical Study in Halobenzene⋯Halobenzene Homodimers

**DOI:** 10.3390/ijms23063114

**Published:** 2022-03-14

**Authors:** Mahmoud A. A. Ibrahim, Rehab R. A. Saeed, Mohammed N. I. Shehata, Muhammad Naeem Ahmed, Ahmed M. Shawky, Manal M. Khowdiary, Eslam B. Elkaeed, Mahmoud E. S. Soliman, Nayra A. M. Moussa

**Affiliations:** 1 Computational Chemistry Laboratory, Chemistry Department, Faculty of Science, Minia University, Minia 61519, Egypt; r.saeed@compchem.net (R.R.A.S.); m.shehata@compchem.net (M.N.I.S.); n.moussa@compchem.net (N.A.M.M.); 2 Department of Chemistry, The University of Azad Jammu and Kashmir, Muzaffarabad 13100, Pakistan; drnaeem@ajku.edu.pk; 3 Science and Technology Unit (STU), Umm Al-Qura University, Makkah 21955, Saudi Arabia; amesmail@uqu.edu.sa; 4 Chemistry Department, Faculty of Applied Science, Umm Al-Qura University, Al-Lith Branch, Makkah 24211, Saudi Arabia; mmkhowdairy@uqu.edu.sa; 5 Department of Pharmaceutical Sciences, College of Pharmacy, AlMaarefa University, Riyadh 13713, Saudi Arabia; ikaeed@mcst.edu.sa; 6 Molecular Modelling and Drug Design Research Group, School of Health Sciences, University of KwaZulu-Natal, Westville, Durban 4000, South Africa

**Keywords:** σ-hole, halogen bond, QTAIM, SAPT-EDA, *trans* → *cis* interconversion

## Abstract

In the current study, unexplored type IV halogen⋯halogen interaction was thoroughly elucidated, for the first time, and compared to the well-established types I–III interactions by means of the second-order Møller–Plesset (MP2) method. For this aim, the halobenzene⋯halobenzene homodimers (where halogen = Cl, Br, and I) were designed into four different types, parodying the considered interactions. From the energetic perspective, the preference of scouted homodimers was ascribed to type II interactions (i.e., highest binding energy), whereas the lowest binding energies were discerned in type III interactions. Generally, binding energies of the studied interactions were observed to decline with the decrease in the σ-hole size in the order, C_6_H_5_I⋯IC_6_H_5_ > C_6_H_5_Br⋯BrC_6_H_5_ > C_6_H_5_Cl⋯ClC_6_H_5_ homodimers and the reverse was noticed in the case of type IV interactions. Such peculiar observations were relevant to the ample contributions of negative-belt⋯negative-belt interactions within the C_6_H_5_Cl⋯ClC_6_H_5_ homodimer. Further, type IV torsional *trans* → *cis* interconversion of C_6_H_5_X⋯XC_6_H_5_ homodimers was investigated to quantify the π⋯π contributions into the total binding energies. Evidently, the energetic features illustrated the amelioration of the considered homodimers (i.e., more negative binding energy) along the prolonged scope of torsional *trans* → *cis* interconversion. In turn, these findings outlined the efficiency of the *cis* configuration over the *trans* analog. Generally, symmetry-adapted perturbation theory-based energy decomposition analysis (SAPT-EDA) demonstrated the predominance of all the scouted homodimers by the dispersion forces. The obtained results would be beneficial for the omnipresent studies relevant to the applications of halogen bonds in the fields of materials science and crystal engineering.

## 1. Introduction

The σ-hole interactions are of growing interest to researchers from various disciplines, owing to their wide applications in catalysis [1], anion recognition [2], biological systems [3,4], crystal materials [5,6], and drug discovery [7]. The occurrence of such interactions was mainly ascribed to the anisotropic distribution of the electron density on the surfaces of group IV-VII elements [8,9,10,11,12,13], resulting in the emergence of an electron depletion region coined as “σ-hole” [14,15]. Among σ-hole interactions, the halogen bond encouraged growing attention by dint of its decisive character in protein–ligand binding affinity [16,17,18], crystal engineering [19,20,21], and organo-catalysis [22,23]. Subsequently, versatile computational [24,25,26,27,28] and experimental [29,30,31,32] studies were devoted to characterizing halogen bonding interactions.

As an essential issue, IUPAC announced the definition of the halogen bond as follows: “A halogen bond occurs when there is evidence of a net attractive interaction between an electrophilic region associated with a halogen atom in a molecular entity and a nucleophilic region in another, or the same, molecular entity” [33]. From literature, the amphoteric nature of the halogen-containing molecules was thoroughly spotted, and their potentiality to simultaneously interact as a Lewis base and a Lewis acid was widely assessed, particularly in the case of like–like complexes [34,35,36,37,38,39,40,41].

Crucially, the ability of halogen-containing molecules to engage in halogen⋯halogen interactions was well-recognized within three different types [35,36,42,43,44]: type I (i.e., parallel displaced geometry): in which the A-halogen–halogen angle (*θ*_1_) is nearly equal to halogen⋯halogen-A angle (*θ*_2_); type II (i.e., *L*-geometry): in which the *θ*_1_ angle is about 180° and *θ*_2_ is about 90°; and type III (i.e., linear geometry), in which the *θ*_1_ and *θ*_2_ angles are typically equal to 180°, where A refers to the attached atom to halogen atom. Further, the literature recorded that the van der Waals, electrostatic, and dispersion forces were the dominant forces beyond type I, II, and III halogen⋯halogen interactions, respectively.

In addition to the three well-established types of halogen⋯halogen interactions, the unconventional *Z*-shaped structure of type IV interactions has recently been well-identified in the previously addressed chalcogen⋯chalcogen homodimers [45]. In this type, the *θ*_1_ = C = Y⋯Y and *θ*_2_ = Y⋯Y = C angles were set to be equal with a value of 90° in the Y = C = Y⋯Y = C = Y (i.e., Y = O, S, and Se) homodimers. Dispersion forces were documented with the most prevalent contributions to the total energy of this type of interactions [45]. Upon thorough literature review, the type IV halogen⋯halogen interactions have not been systematically elucidated yet.

**Figure 1:**
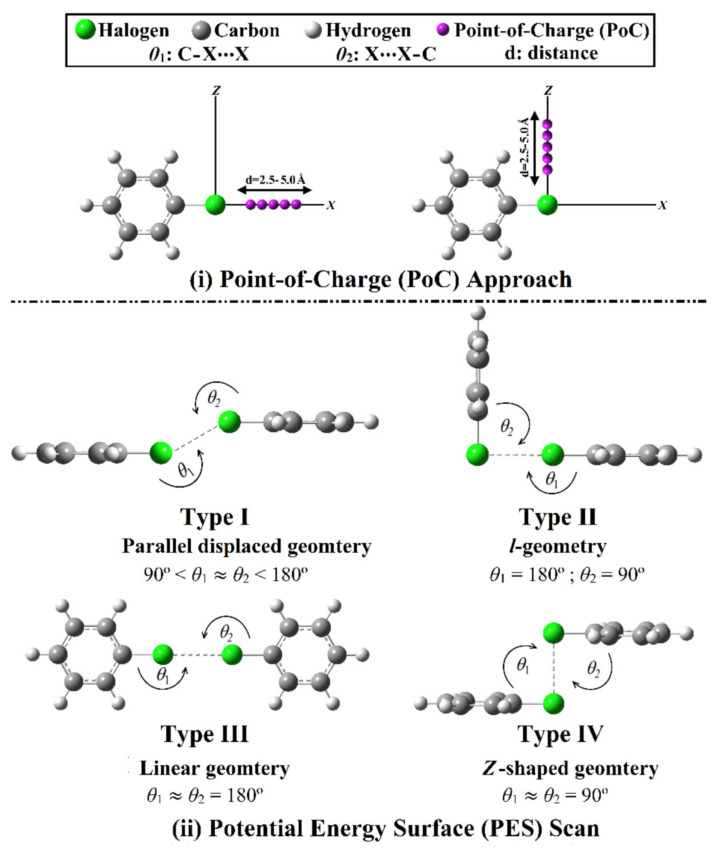
Schematic representation for (**i**) the point-of-charge (PoC) calculations for halobenzene monomers (C_6_H_5_X; where X = Cl, Br, and I); and (**ii**) the potential energy surface (PES) scan for the designed halogen⋯halogen interactions within the C_6_H_5_X⋯XC_6_H_5_ homodimers.

Therefore, the current study was devoted to comparatively assessing the potentiality of halogen-containing molecules (C_6_H_5_X; where X = Cl, Br, and I) to participate in type I–IV halogen⋯halogen interactions within C_6_H_5_X⋯XC_6_H_5_ homodimers (Figure 1). Geometrical optimization, electrostatic potential (EP) analysis, and point-of-charge (PoC) approach calculations were carried out on the studied monomers. For the C_6_H_5_X⋯XC_6_H_5_ homodimers, a potential energy surface (PES) scan was performed in an exact orientation to obtain the four modeled types of interaction. Furthermore, quantum theory of atoms in molecules (QTAIM), noncovalent interaction (NCI) index analyses, and symmetry adapted perturbation theory-based energy decomposition analysis (SAPT-EDA) were adapted to elucidate the nature of the considered interactions. Such observations would be an opulent tool for identifying the complete nature of halogen⋯halogen interactions and their roles in supramolecular chemistry and materials science fields.

## 2. Results

### 2.1. Electrostatic Potential (EP) Analysis

**Figure 2:**
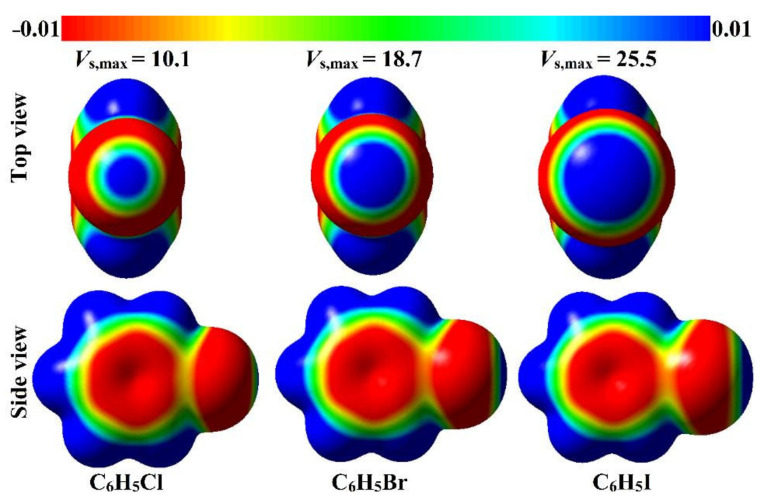
Molecular electrostatic potential (MEP) maps of the halobenzene (i.e., C_6_H_5_X, where X = Cl, Br, and I) are plotted using 0.002 electron density contours. The electrostatic potential varies from −0.01 (red) to +0.01 (blue) au. The maximum positive electrostatic potentials (*V*_s,max_) at σ-hole are given in kcal/mol.

Electrostatic potential (EP) analysis was recently deemed as an informative appliance to precisely scrutinize the electrophilicity and nucleophilicity over the molecular surface of the optimized monomers [46,47]. Thus, molecular electrostatic potential (MEP) maps were generated for the C_6_H_5_X molecules (where X = Cl, Br, and I) to graphically identify the electron-depletion and electron-rich regions. Additionally, maximum positive electrostatic potential (*V*_s,max_) calculations were executed to quantify the maximum positive EP regions for all the studied molecules. MEP maps and *V*_s,max_ values of the considered monomers are displayed in Figure 2.

From the MEP maps depicted in Figure 2, the prominent blue regions were disclosed with variable sizes along the molecular surface of the halogen atom in the studied systems, outlining the occurrence of σ-holes. Conspicuously, the σ-hole size was perceived to increase in the order Cl < Br < I, according to increasing the atomic size of the halogen atom. The posterior results were ascribed to the ascending of the polarizability along with the lesser electronegativity of the heavier halogen atoms. From a quantitative perspective, the obtained σ-hole magnitude using *V*_s,max_ analysis was observed to be consistent with the σ-hole size pictured in MEP maps. Numerically, a positive EP region was detected with values of 10.1, 18.7, and 25.5 kcal/mol for C_6_H_5_Cl, C_6_H_5_Br, and C_6_H_5_I, respectively. These results were found to be in agreement with the previous findings [43].

### 2.2. Point-of-Charge (PoC) Calculations

The Point-of-charge (PoC) approach was documented as an informative tool for elucidating the electrostatic inclination of the group III-VIII element-containing molecules to participate in noncovalent interactions [48,49,50,51,52,53,54,55,56]. With the help of the PoC approach, negatively and positively charged points were utilized to parody the nucleophilic and electrophilic effects on the inspected molecular systems, respectively. In that context, molecular stabilization energy for C_6_H_5_X⋯PoC systems (where X = Cl, Br, and I) were estimated using values of ±0.50 au PoCs at a distance range of 2.5–5.0 Å with a step size of 0.1 Å along the *x*-axis and *z*-axis of the halogen atoms (i.e., the equatorial and axial surfaces) with ∠C-X⋯PoC angle of 180° and 90°, respectively.

**Figure 3:**
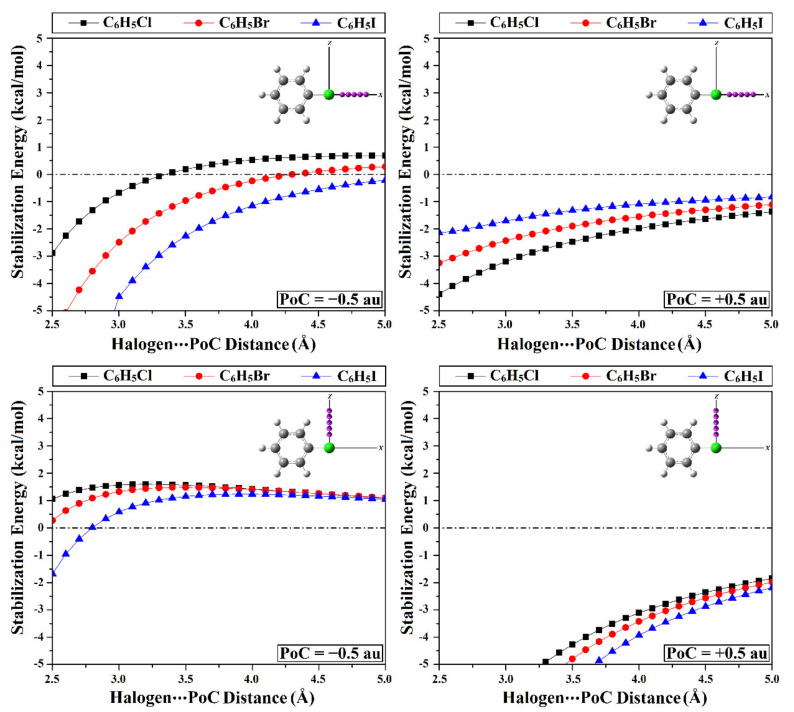
Molecular stabilization/destabilization energies (*E*_stabilization_/*E*_destabilization_) of the C_6_H_5_X⋯PoC systems (where X = Cl, Br, and I) in the presence of ±0.50 au PoCs at X⋯PoC distance ranging from 2.5 to 5.0 Å along the *x*-axis and *z*-axis with ∠C-X⋯PoC angle of 180° and 90°, respectively.

As delineated in Figure 3, molecular stabilization energy curves generally outlined the predilection of most of the study’s halobenzene (C_6_H_5_X) monomers to engage in electrostatic interactions with negative and positive PoCs (i.e., the Lewis base and acid) along both *x*- and *z*-axes. Notably, all the studied C_6_H_5_X⋯PoC systems bolstered the nucleophilic effects (i.e., negative PoC) along the equatorial portions of halogen atoms (i.e., *x*-axis) over the outermost axial surface (i.e., *z*-axis), and the reverse was true for the electrophilic effects.

Regarding the negative PoC-based results, all the investigated C_6_H_5_X⋯PoC systems showed molecular stabilization energy (*E*_stabilization_) in the presence of −0.50 au PoC along the *x*-axis. In contrast, molecular destabilization energy (*E*_destabilization_) was recorded for all systems along the *z*-axis with an exception for the C_6_H_5_I molecule, which exhibited favorable *E*_stabilization_ with a value of −1.68 kcal/mol (Table 1). Obviously, the molecular stabilization energies of C_6_H_5_X⋯PoC systems were perceived to increase coinciding with the atomic size of the halogen atom as follows: C_6_H_5_Cl⋯ < C_6_H_5_Br⋯ < C_6_H_5_I⋯PoC systems. As an illustration, *E*_stabilization_/*E*_destabilization_ values were –2.88, –6.08, and –10.99 kcal/mol along the *x*-axis and 1.06, 0.28, and –1.68 kcal/mol along the *z*-axis for C_6_H_5_Cl⋯, C_6_H_5_Br⋯, C_6_H_5_I⋯PoC systems, respectively.

Turning to the positive PoC-based results, molecular stabilization energies were disclosed to decrease along the outermost equatorial surface according to the following order, C_6_H_5_Cl⋯ > C_6_H_5_Br⋯ > C_6_H_5_I⋯PoC. Evidently, an inverse correlation was obtained between *E*_stabilization_ and the halogen atomic size that might be ascribed to the elevated σ-hole size of the halogen atom. Contrarily, along the axial surface, *E*_stabilization_ was perceived to increase with decreasing the electronegativity of the halogen atoms. For instance, molecular stabilization energies were detected with values of –4.39, –3.25, and –2.14 kcal/mol along the *x*-axis accompanied by *E*_stabilization_ of –9.70, –11.31, and –13.54 kcal/mol along the *z*-axis of halogens of the C_6_H_5_Cl⋯, C_6_H_5_Br⋯, and C_6_H_5_I⋯PoC systems, respectively. It is worth mentioning that the PoC results obtained for C_6_H_5_X⋯PoC systems along the *x*-axis are consistent with a previous study in exhibiting direct and inverse correlations with the negative molecular stabilization energy in the presence of negative and positive PoCs, respectively [43].

### 2.3. Potential Energy Surface (PES) Scan

**Figure 4:**
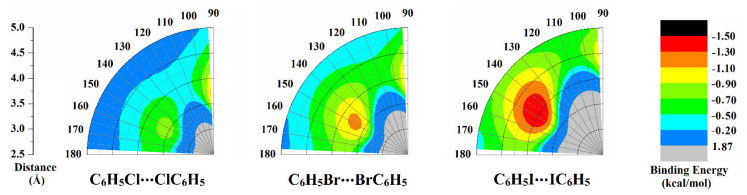
Colored binding energy (*E*_binding_) maps of type I halogen⋯halogen interactions within the C_6_H_5_X⋯XC_6_H_5_ homodimers (where X = Cl, Br, and I) at X⋯X distance range of 2.5–5.0 Å and angle range of 90° < *θ*_1_ ≈ *θ*_2_ < 180°.

**Figure 5:**
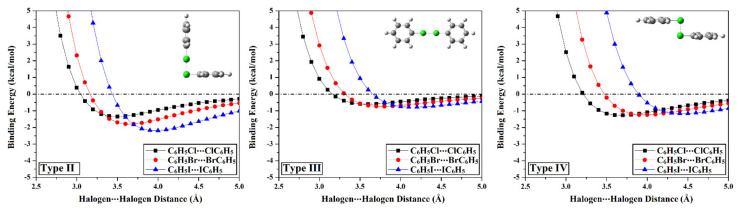
Binding energy curves for type II, III, and IV halogen⋯halogen interactions within the C_6_H_5_X⋯XC_6_H_5_ homodimers (where X = Cl, Br, and I) estimated at MP2/aug–cc–pVDZ(PP) level of theory in kcal/mol at X–X distance range of 2.5–5.0 Å.

A potential energy surface (PES) scan was executed to thoroughly elucidate the ability of the C_6_H_5_X monomers (where X = Cl, Br, and I) to engage in type I–IV halogen⋯halogen interactions. PES scan was implemented for the C_6_H_5_X⋯XC_6_H_5_ homodimers at an X⋯X distance range of 2.5–5.0 Å with a step size of 0.1 Å. Colored binding energy (*E*_binding_) maps were conceived to establish an in-depth insight into type I halogen⋯halogen interactions with a ∠C-X⋯X of 90° < *θ*_1_ ≈ *θ*_2_ < 180° (Figure 4). For type II, III, and IV halogen⋯halogen interactions, the binding energy curves were generated and are displayed in Figure 5. The MP2/aug–cc–pVDZ(PP) binding energies were assessed at the most favorable X⋯X distance and are listed along with the complexation parameters of type I–IV halogen⋯halogen interactions in Table 2. The coordinates of the most preferential complexes are collected in Table S1.

From the colored maps illustrated in Figure 4, binding energies of type I interactions increased with enlarging the σ-hole size of halogen atoms in the order C_6_H_5_Cl⋯ClC_6_H_5_ < C_6_H_5_Br⋯BrC_6_H_5_ < C_6_H_5_I⋯IC_6_H_5_ homodimers. For example, the binding energies of the C_6_H_5_X⋯XC_6_H_5_ homodimers were –1.12, –1.15, and –1.49 kcal/mol in the case of X = Cl, Br, and I, respectively. The abovementioned pattern of the energetic features was in outstanding consistency with previously reported results, in which enhancement of the strength of type I halogen⋯halogen interactions was observed in the same order [57].

For C_6_H_5_Cl⋯ClC_6_H_5_ homodimer, the binding energy was found to generally increase by decreasing *θ*_1_ and *θ*_2_ until a certain angle of *θ*_1_ = *θ*_2_ = 147.5°. After that point, the *E*_binding_ fluctuated by decreasing the *θ*_1_ and *θ*_2_ values from 115° to 92.5°. For instance, binding energies were –0.58, –0.73, –0.36, and –1.12 in the case of the diminishing of *θ*_1_ and *θ*_2_ values from 177.5° to 147.5°, 115°, and 92.5°, respectively (Table 2). Meanwhile towering *E*_binding_ was disclosed for the C_6_H_5_Br⋯BrC_6_H_5_ and C_6_H_5_I⋯IC_6_H_5_ homodimers by decreasing *θ*_1_ and *θ*_2_ till it recorded its maxima at *θ*_1_ = *θ*_2_ = 147.5° and then decreased with diminishing *θ*_1_ and *θ*_2_ values. As an illustration, the *E*_binding_ of the C_6_H_5_I⋯IC_6_H_5_ homodimer was –0.76, –1.49, and –0.27 kcal/mol at *θ*_1_ = *θ*_2_ = 177.5°, 147.5°, and 92.5°, respectively. Conspicuously, these results illuminated the prevalence of negative-belt⋯negative-belt interactions within C_6_H_5_Cl⋯ClC_6_H_5_ homodimer (i.e., almost more favorable *E*_binding_) as *θ*_1_ and *θ*_2_ became closer to 92.5°, relatively in comparison to the C_6_H_5_Br⋯BrC_6_H_5_ and C_6_H_5_I⋯IC_6_H_5_ counterparts.

Apparently, binding energies of type II interactions were spotted to drastically increase with the growth in the atomic size of the interacting halogen atoms, which was previously reported and confirmed by the obtained values [58]. Numerically, *E*_binding_ was estimated with values of –1.32, –1.80, and –2.20 kcal/mol for C_6_H_5_Cl⋯ClC_6_H_5_, C_6_H_5_Br⋯BrC_6_H_5_, and C_6_H_5_I⋯IC_6_H_5_ homodimers, respectively.

With respect to type III interactions, as an upshot to the presence of σ-hole along the molecular surface of the scouted molecules, specific directional bonding interactions between the two identically charged halogens were divulged, dubbed as σ-hole⋯σ-hole interactions [43]. The σ-hole⋯σ-hole interactions were discerned to debilitate with decreasing the σ-hole size (i.e., increasing the electronegativity) of the halogen atom that was in line with our previous work [43]. For instance, the *E*_binding_ of σ-hole⋯σ-hole interactions was assessed with values of –0.79, –0.74, and –0.60 kcal/mol for C_6_H_5_I⋯IC_6_H_5_, C_6_H_5_Br⋯BrC_6_H_5_, and C_6_H_5_Cl⋯ClC_6_H_5_ homodimers, respectively. Such observations might be interpreted as a consequence of the contributions of the polarization of one of the interacted halogen atoms by the other one.

Regarding unexplored type IV halogen⋯halogen interactions, a direct correlation was noticed between the binding energy and the atomic size of the halogen atoms, exhibiting the following order C_6_H_5_Cl⋯ClC_6_H_5_ > C_6_H_5_Br⋯BrC_6_H_5_ > C_6_H_5_I⋯IC_6_H_5_ homodimers. Evidently, binding energies of C_6_H_5_X⋯XC_6_H_5_ homodimers were detected with values of –1.27, –1.25, and –1.16 kcal/mol when X = Cl, Br, and I, respectively. These unconventional findings might be ascribed to the resurgent effect of negative-belt⋯negative-belt interactions on the *E*_binding_ of the studied homodimers. Hence, type IV-based results could be considered as an integrative window to the foregoing analog of type I interactions at *θ*_1_ = *θ*_2_ = 92.5°.

Overall, all the inspected halogen-containing molecules showed a propensity to participate in type I–IV halogen⋯halogen interactions. Clearly, the energetic features outlined that the highest binding energy was discerned to type II interactions, which is inconsistent with the chalcogen⋯chalcogen analogs that announced the preference of type IV interactions [53]. On the other hand, the lowest *E*_binding_ was relevant to type III interactions synchronically to chalcogen⋯chalcogen counterparts.

### 2.4. Quantum Theory of Atom in Molecules (QTAIM) Analysis

**Figure 6:**
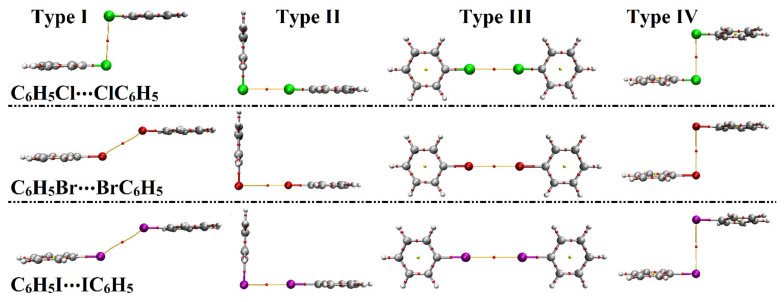
Quantum theory of atoms in molecules (QTAIM) diagrams for C_6_H_5_X⋯XC_6_H_5_ homodimers (where X = Cl, Br, and I) in the fashion of type I–IV halogen⋯halogen interactions at the most favorable parameters. The gray, white, green, red, and purple colored balls represent the carbon, hydrogen, chlorine, bromine, and iodine atoms, respectively.

Quantum theory of atom in molecules (QTAIM), proclaimed by Bader et al., has been deemed as an authoritative tool to give a thorough foresight into the nature of intermolecular interactions [59]. For the inspected homodimers, QTAIM analysis was accomplished to uncover the occurrence of the halogen⋯halogen interactions by generating bond critical points (BCPs) and bond paths (BPs). Within the context of QTAIM, diverse BCP features, including electron density (*ρ*_b_), Laplacian (∇^2^*ρ*_b_), and total energy density (H_b_), were minutely estimated for the C_6_H_5_X⋯XC_6_H_5_ homodimers at the most favorable parameters. The BPs and BCPs were generated and are depicted in Figure 6. The obtained electron density (*ρ*_b_), Laplacian (∇^2^*ρ*_b_), and total energy density (H_b_) are listed in Table 3.

As displayed in Figure 6, the potentiality of the scouted monomers to engage in halogen⋯halogen interactions was clearly affirmed through the presence of one bond path (BP) and bond critical point (BCP) between the interacting species. From the data listed in Table 3, positive values of electron density (*ρ*_b_), Laplacian (∇^2^*ρ*_b_), and total energy density (H_b_) were observed, reflecting the closed-shell nature of the considered interactions. Obviously, the *ρ*_b_, ∇^2^*ρ*_b_, and H_b_ values were generally observed to be in coincidence with the energetic features. For instance, the *ρ*_b_ was estimated for type IV interactions with values of 0.00603, 0.00516, and 0.00439 au, along with binding energies of –1.27, –1.25, and –1.16 kcal/mol for C_6_H_5_Cl⋯ClC_6_H_5_, C_6_H_5_Br⋯BrC_6_H_5_, and C_6_H_5_I⋯IC_6_H_5_ homodimers, respectively.

### 2.5. Noncovalent Interactions (NCI) Analysis

**Figure 7:**
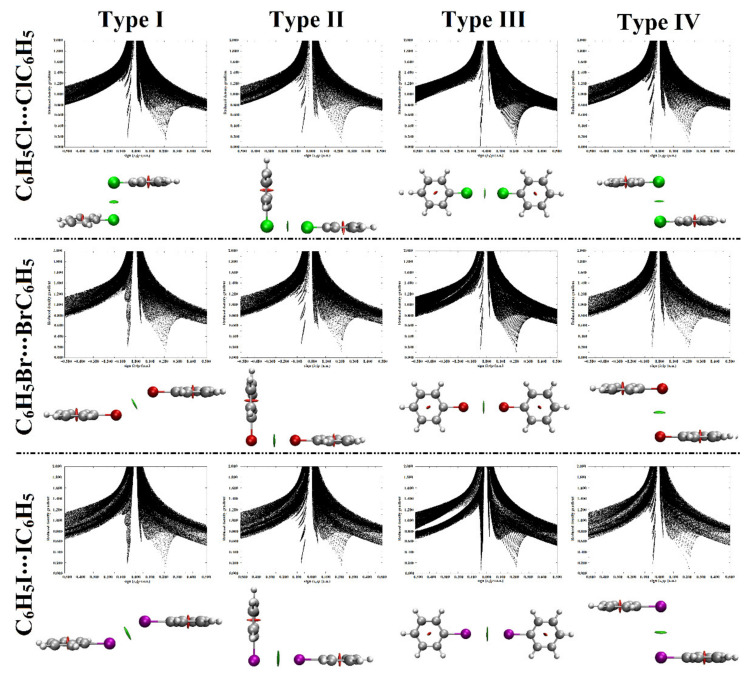
2D and 3D noncovalent interaction (NCI) isosurfaces for C_6_H_5_X⋯XC_6_H_5_ homodimers (where X = Cl, Br, and I) in the pattern of type I–IV halogen⋯halogen interactions at the most favorable parameters. The isosurfaces were plotted with a reduced density gradient value of 0.50 au and colored from blue to red according to sign(λ_2_)*ρ* ranging from −0.035 (blue) to 0.020 (red) au. The gray, white, green, red, and purple colored balls represent the carbon, hydrogen, chlorine, bromine, and iodine atoms, respectively.

The Noncovalent Interactions (NCI) index was endorsed to be a trustworthy method for providing a qualitative view of weak intermolecular interaction based on the electron density and its derivatives [60]. The 2D reduced density gradient (RDG) versus the electron density (*ρ*) multiplied by the sign of the second Hessian eigenvalue (λ_2_) were generated for the studied homodimers. Besides, the 3D NCI plots, with a color scale of sign(λ_2_)*ρ* from −0.035 (blue) to 0.020 (red), where λ_2_ is the second eigenvalue of the Hessian matrix and *ρ* is the electron density, were then extracted. The obtained 2D and 3D NCI plots are shown in Figure 7.

According to Figure 7, green isosurfaces were observed within the C_6_H_5_X⋯XC_6_H_5_ homodimers, affirming the penchant of the considered halogen-containing molecules to participate in type I–IV halogen⋯halogen interactions. Moreover, the corresponding spikes of sign (λ_2_)*ρ* at low densities emphasized the existence of attractive interactions (sign (λ_2_)*ρ* < 0). From the synchronic perspective to the MP2 energetic features, the size of the green-coded regions and the shifting magnitude of the spikes towards the negative sign (λ_2_)*ρ* were disclosed to be in line with the binding energy results registered in Table 2. Overall, the results of the QTAIM and NCI index analyses support previous studies that illustrated the interactions within the type II and III halogen⋯halogen complexes [43,58].

### 2.6. Symmetry-Adapted Perturbation Theory-Based Energy Decomposition Analysis (SAPT-EDA)

**Figure 8:**
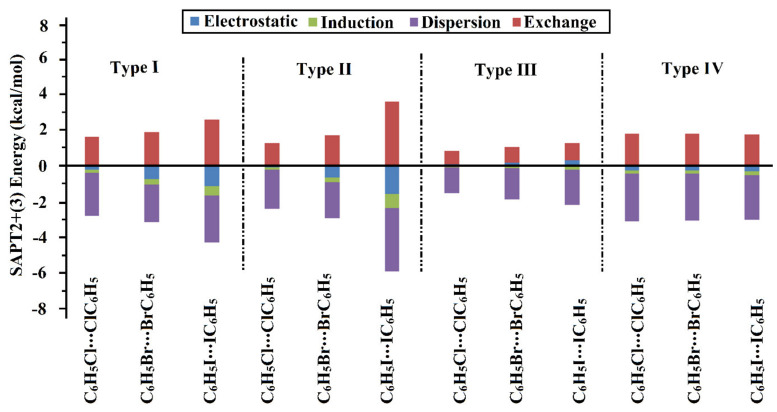
Bar chart showed the physical components of total SAPT2+(3) energy, including electrostatic (*E*_elst_), induction (*E*_ind_), dispersion (*E*_disp_), and exchange (*E*_exch_) terms, for C_6_H_5_X⋯XC_6_H_5_ homodimers (where X = Cl, Br, and I) in the fashion of type I–IV halogen⋯halogen interactions at the most favorable parameters.

Symmetry-adapted perturbation theory-based energy decomposition analysis (SAPT-EDA) was fulfilled toward a further illustration of the physical nature of type I–IV halogen⋯halogen interactions. Using SAPT-EDA, the total binding energy was decomposed into its fundamental physical components, namely electrostatic (*E*_elst_), induction (*E*_ind_), dispersion (*E*_disp_), and exchange (*E*_exch_). SAPT-EDA calculations were performed for all of the four types of halogen⋯halogen interactions at the SAPT2+(3) level of truncation [61] using PSI4 code [62]. Total SAPT2+(3) energy accompanied with its physical components is collected in Table 4 and shown on a graph in Figure 8.

As shown in Figure 8, the dispersion energy (*E*_disp_) was the predominant force within all of the scouted types of halogen ⋯halogen interactions. Moreover, respectable contributions for the electrostatic (*E*_elst_) and induction (*E*_ind_) interactions were obtained in the four considered types of halogen⋯halogen interactions. On the other hand, unfavorable contributions for the exchange energy (*E*_exch_) were estimated with positive values for all homodimers. For instance, in the case of type IV interaction for C_6_H_5_Cl⋯ClC_6_H_5_ homodimer, *E*_elst_, *E*_ind_, *E*_disp_, and *E*_exch_ were assessed with values of −0.28, −0.18, −2.67, and 1.83 kcal/mol, respectively (Table 4).

From the data listed in Table 4, the electrostatic (*E*_elst_), induction (*E*_ind_), and dispersion (*E*_disp_) forces were generally observed to be in coincidence with the MP2 energetic features. For example, the *E*_disp_ of type IV interactions were evaluated with values of –2.67, –2.65, and –2.54 kcal/mol accompanied with binding energies of –1.27, –1.25, and –1.16 kcal/mol for C_6_H_5_Cl⋯ClC_6_H_5_, C_6_H_5_Br⋯BrC_6_H_5_, and C_6_H_5_I⋯IC_6_H_5_ homodimers, respectively. Particularly, for σ-hole⋯σ-hole interactions (i.e., type III interactions), unfavorable *E*_elst_ was detected, that was observed to escalate with increasing the σ-hole size along with the molecular surface of the halogen atoms, as previously documented [43]. Evidently, *E*_elst_ were 0.09, 0.17, and 0.32 kcal/mol for C_6_H_5_Cl⋯ClC_6_H_5_, C_6_H_5_Br⋯BrC_6_H_5_, and C_6_H_5_I⋯IC_6_H_5_ homodimers, respectively. The precision of the employed level for the SAPT-EDA calculations was affirmed by quantifying the energy difference between the MP2 binding energy and the computed SAPT2+(3) energy (∆∆*E*) with nominal values.

### 2.7. π⋯π. Contribution into Type IV Halogen⋯Halogen Interaction

**Figure 9:**
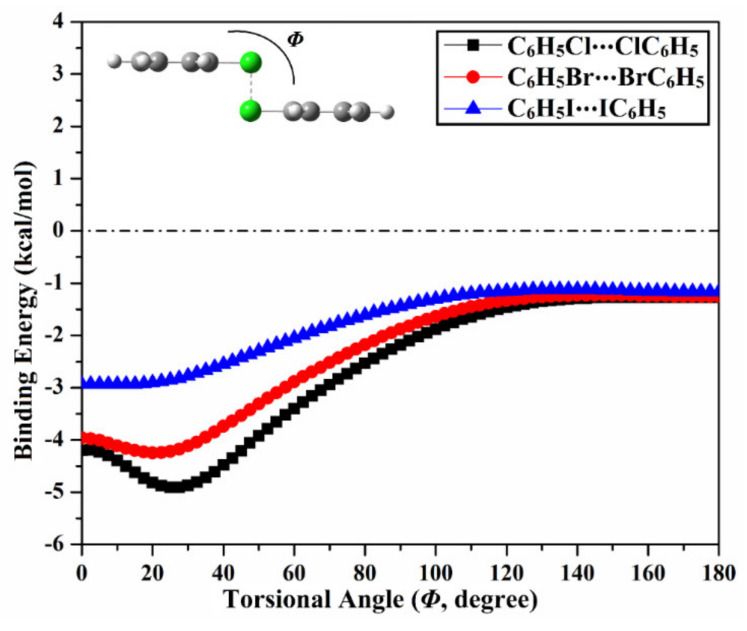
Binding energy curves for torsional *trans → cis* interconversion of C_6_H_5_X⋯XC_6_H_5_ homodimers (where X = Cl, Br, and I) in the pattern of type IV halogen⋯halogen interactions at the most favorable parameters.

To gain a deep insight into π–π contributions within type IV halogen⋯halogen interactions, torsional *trans* → *cis* interconversion of C_6_H_5_X⋯XC_6_H_5_ homodimers (where X = Cl, Br, and I) were investigated (Figure 9). In this context, the torsional *trans* → *cis* interconversion was acclimated by scanning the dihedral angle *Φ* (C-X⋯X-C) in the range from 180º (i.e., *trans* configuration) to 0° (i.e., *cis* configuration) with an interval of 2.5°. Binding energy curves of torsional *trans* → *cis* interconversion of the inspected homodimers are illustrated in Figure 9. The negative energetic values for the *trans* and *cis* configurations and the maximum binding energy (*E*_max_) within the prolonged range of the torsional *trans* → *cis* interconversion were estimated and are listed in Table 5.

Looking at Figure 9, torsional *trans → cis* interconversion provided a privileged deal for all the inspected homodimers, in which the towering binding energies (i.e., more negative) were generally observed ongoing from *Φ* = 180° (i.e., *trans* configuration) to *Φ* = 0° (i.e., *cis* configuration). Evidently, binding energy was found to augment from –1.27, –1.25, –1.16 kcal/mol at *Φ* =180° till recorded at its maxima at *Φ =* 25°, 20°, and 10° with values of –4.91, –4.25, and –2.93 kcal/mol for C_6_H_5_Cl⋯ClC_6_H_5_, C_6_H_5_Br⋯BrC_6_H_5_, and C_6_H_5_I⋯IC_6_H_5_, respectively. Turning to the *cis* configuration, the binding energies were found to be increased according to the following order C_6_H_5_Cl⋯ClC_6_H_5_ > C_6_H_5_Br⋯BrC_6_H_5_ > C_6_H_5_I⋯IC_6_H_5_ with values of –4.20, –3.96, and –2.93 kcal/mol, respectively, coinciding with the *trans* configuration considerations. Astonishingly, the proceeding results accentuated that the *cis* configuration was outshined by the *trans* configuration (i.e., type IV interactions), highlighting the paramount contributions of the π–π interactions.

## 3. Computational Methods

In the presented study, the inclination of halobenzene molecules (C_6_H_5_X; where X = Cl, Br, and I) to participate in type I–IV halogen⋯halogen interactions were comparatively scrutinized. Geometrical structures of the investigated monomers were fully optimized at the MP2/aug–cc–pVDZ level of theory [63,64] for all the studied atoms except Br and I atoms that were treated with aug–cc–pVDZ(PP) basis set to consider the relativistic effects [65]. Upon the optimized monomers, the electrostatic potential (EP) analysis was carried out using an electron density contour of 0.002 au as a precious envelop that described the electrostatic potential of the chemical systems [7,36]. In that spirit, the MEP maps were generated, and the maximum positive electrostatic potential (*V*_s,max_) values were computed with the help of Multiwfn 3.7 software [66]. Using the point-of-charge (PoC) approach, the propensity of the studied monomers to engage in type I-V halogen⋯halogen interactions from the electrostatic perspective was preciously appreciated [58,67,68,69]. In the PoC approach, negatively- and positively charged points with a value of ±0.50 au were employed to simulate the interactions of halobenzene with a Lewis base and acid, respectively. Accordingly, molecular stabilization energy (*E*_stabilization_) was assessed at X–PoC distance ranging from 2.5 to 5.0 Å with a step size of 0.1 Å and ∠C-X–PoC angle of 180° and 90° along the *x*-axis and *z*-axis of the halogen atom (i.e., along the equatorial and axial surfaces of the halogen atom), respectively. Molecular stabilization energy (*E*_stabilization_) of the C_6_H_5_⋯PoC systems was calculated according to the following equation [34,52,67,70,71]:

(1) $$E_{\mathrm{stabilization}}=E_{\mathrm{halogen}-\mathrm{containing}\;\mathrm{molecule}\cdots \mathrm{PoC}}-E_{\mathrm{halogen}-\mathrm{containing}\;\mathrm{molecule}}$$

A potential energy surface (PES) scan was also implemented to elucidate the potentiality of the C_6_H_5_X⋯XC_6_H_5_ homodimers to participate in type I–IV halogen⋯halogen interactions. The optimized monomers were directed through a set of exact orientations parodying the studied interaction types without allowing the optimized monomers to be distorted. PES scan was performed for the considered homodimers at X–X distance range of 2.5–5.0 Å with a step size of 0.1 Å. Binding energy was then calculated as the difference between the energy of the homodimer and the sum of its monomers. The basis set superposition error (BSSE) was eliminated from the computed binding energies using the counterpoise correction (CP) method [72].

Quantum theory of atoms in molecules (QTAIM) was invoked to qualitatively elaborate the nature of the studied homodimers [73]. In the context of QTAIM, the bond paths (BPs) and bond critical points (BCPs) were generated. Various topological features, including electron density (*ρ*_b_), Laplacian (∇^2^*ρ*_b_) and total energy density (H_b_), were also evaluated. Noncovalent interaction (NCI) index was then fulfilled to get a deeper pictorial insight into the nature of the considered interactions in the fashion of 2D-reduced density gradient (RDG) and 3D-colored NCI plots [60]. The QTAIM and NCI analyses were performed using Multiwfn 3.7 package [66] and portrayed using Visual Molecular Dynamics (VMD) software [74]. All calculations were executed at the MP2/aug–cc–pVDZ(PP) level of theory using Gaussian 09 software [75].

Symmetry-adapted perturbation theory-based energy decomposition analysis (SAPT-EDA) was incorporated as a robust tool to decompose the binding energy into its fundamental physical components. SAPT-EDA calculations were performed for the studied homodimers at SAPT2+(3) level of truncation [61] with the help of the PSI4 code [62]. The total SAPT2+(3) energy components, involving electrostatic (*E*_elst_), induction (*E*_ind_), dispersion (*E*_disp_), and exchange energies (*E*_exch_) were computed according to the following equations [76]:

(2) $$E_{\mathrm{SAPT}2+\left(3\right)}^{}=E_{\mathrm{elst}}+E_{\mathrm{ind}}+E_{\mathrm{disp}}+E_{\mathrm{exch}}$$

where:

(3) $$E_{\mathrm{elst}}=E_{\mathrm{elst}}^{\left(10\right)}+E_{\mathrm{elst},r}^{\left(12\right)}+E_{\mathrm{elst},r}^{\left(13\right)}$$

(4) $$E_{\mathrm{ind}}=E_{\mathrm{ind},r}^{\left(20\right)}+E_{\mathrm{exch}-\mathrm{ind},r}^{\left(20\right)}+E_{\mathrm{ind}}^{\left(22\right)}+E_{\mathrm{exch}-\mathrm{ind}}^{\left(22\right)}+\delta E_{\mathrm{HF},r}^{\left(2\right)}$$

(5) $$E_{\mathrm{disp}}=E_{\mathrm{disp}}^{\left(20\right)}+E_{\mathrm{exch}-\mathrm{disp}}^{\left(20\right)}+E_{\mathrm{disp}}^{\left(21\right)}+E_{\mathrm{disp}}^{\left(22\right)}\left(\mathrm{SDQ}\right)+E_{\mathrm{Est}.\;\mathrm{Disp}\;}^{\left(22\right)}\left(T\right)+E_{\mathrm{disp}}^{\left(30\right)}$$

(6) $$E_{\mathrm{exch}}=E_{\mathrm{exch}}^{\left(10\right)}+E_{\mathrm{exch}}^{\left(11\right)}+E_{\mathrm{exch}}^{\left(12\right)}$$

## 4. Conclusions

In this study, four different types of halogen⋯halogen interactions were comparatively scrutinized within the C_6_H_5_X⋯XC_6_H_5_ homodimers (where X = Cl, Br, and I), using a versatile range of quantum mechanical calculations. The occurrence of the σ-hole along the surface of the studied halogen atoms was found to be the common feature within the inspected molecules, as affirmed by MEP maps, which enabled them to behave as potent halogen bond donors. Astonishingly, PoC calculations generally assured the penchant of the considered molecules to electrostatically engage in halogen bonding interactions along the *x*- and *z*-axes. For the inspected homodimers, towering binding energies were evidently recorded with disparate magnitudes, alluding to the significant propensity of the scouted molecules to engage in type I–IV halogen⋯halogen interactions. Interestingly, for the explored interactions, type II interactions were reported as the superb geometry with favorable negative energies. In line with chalcogen⋯chalcogen interactions, type III halogen⋯halogen interactions were denoted as the most emaciated one. Generally, binding energy was noticed to increase coinciding with the atomic size of the halogen atoms. In particular, an alien behavior was uncovered within type IV interactions, in which an inversely proportional correlation was observed between the *E*_binding_ and the σ-hole size as an upshot to the varied negative-belt⋯negative-belt contributions. Moreover, the prolonged scope of the torsional *trans* → *cis* interconversion interactions was reported with favorable energetic values that was found to be in consistence with type IV interactions based-results for all the considered homodimers. SAPT-EDA results addressed the dispersion forces as the most preferential component within all the four studied interaction types. The full insight into the type I–IV halogen⋯halogen interactions that emerged from the current work would be fundamental guidance for the forthcoming studies pertinent to the crystal engineering and materials science fields.

## Figures and Tables

**Table 1:** Molecular stabilization/destabilization energies (*E*_stabilization_/*E*_destabilization_) of the C_6_H_5_X⋯PoC systems (where X = Cl, Br, and I) in the presence of ±0.50 au PoC at X⋯PoC distance of 2.5 Å along the *x*-axis and *z*-axis with ∠C-X⋯PoC angle of 180° and 90°, respectively.

System	Molecular Stabilization Energy (*E*_stabilization_/*E*_destabilization_, kcal/mol)			
	PoC= −0.50 au		PoC= +0.50 au	
	*x*-Axis	*z*-Axis	*x*-Axis	*z*-Axis
C_6_H_5_Cl⋯PoC	−2.88	1.06	−4.39	–9.70
C_6_H_5_Br⋯PoC	−6.08	0.28	−3.25	–11.31
C_6_H_5_I⋯PoC	−10.99	−1.68	−2.14	–13.54

**Table 2:** Binding energies calculated (in kcal/mol) at MP2/aug–cc–pVDZ(PP) level of theory for C_6_H_5_X⋯XC_6_H_5_ homodimers (where X = Cl, Br, and I) via type I–IV halogen⋯halogen interactions at the most favorable parameters.

Homodimer		Complexation Parameters ^a^			Binding Energy
		Distance (Å)	*θ*_1_^b^ (Degree)	*θ*_2_^b^ (Degree)	*E*_MP2/aug−cc−pVDZ_ (kcal/mol)
Type I	C_6_H_5_Cl⋯ClC_6_H_5_	3.71	92.5°	92.5°	–1.12
	C_6_H_5_Br⋯BrC_6_H_5_	3.71	147.5°	147.5°	–1.15
	C_6_H_5_I⋯IC_6_H_5_	4.02	147.5°	147.5°	–1.49
Type II	C_6_H_5_Cl⋯ClC_6_H_5_	3.49	90°	180°	–1.32
	C_6_H_5_Br⋯BrC_6_H_5_	3.64			–1.80
	C_6_H_5_I⋯IC_6_H_5_	3.96			–2.20
Type III	C_6_H_5_Cl⋯ClC_6_H_5_	3.58	180°	180°	–0.60
	C_6_H_5_Br⋯BrC_6_H_5_	3.77			–0.74
	C_6_H_5_I⋯IC_6_H_5_	4.15			–0.79
Type IV	C_6_H_5_Cl⋯ClC_6_H_5_	3.68	90°	90°	–1.27
	C_6_H_5_Br⋯BrC_6_H_5_	3.97			–1.25
	C_6_H_5_I⋯IC_6_H_5_	4.43			–1.16

^a^ the most favorable parameters based on the binding energy maps and curves in Figure 4 and Figure 5, respectively; ^b^ *θ*_1_ and *θ*_2_ represented the ∠C-X⋯X and ∠X⋯X-C bond angles, respectively.

**Table 3:** Topological parameters, including the electron density (*ρ*_b_, au), Laplacian (∇^2^*ρ*_b_, au), and total energy density (H_b_, au), at bond critical points (BCPs) for C_6_H_5_X⋯XC_6_H_5_ homodimers (where X = Cl, Br, and I) in the fashion of type I–IV halogen⋯halogen interactions at the most favorable parameters.

	Homodimer	*ρ*_b_ (au)	∇^2^*ρ*_b_ (au)	H_b_ (au)
Type I	C_6_H_5_Cl⋯ClC_6_H_5_	0.00573	0.01575	0.00050
	C_6_H_5_Br⋯BrC_6_H_5_	0.00584	0.01839	0.00092
	C_6_H_5_I⋯IC_6_H_5_	0.00604	0.01499	0.00064
Type II	C_6_H_5_Cl⋯ClC_6_H_5_	0.00660	0.02206	0.00091
	C_6_H_5_Br⋯BrC_6_H_5_	0.00708	0.02255	0.00107
	C_6_H_5_I⋯IC_6_H_5_	0.00713	0.01773	0.00072
Type III	C_6_H_5_Cl⋯ClC_6_H_5_	0.00417	0.01567	0.00077
	C_6_H_5_Br⋯BrC_6_H_5_	0.00406	0.01470	0.00077
	C_6_H_5_I⋯IC_6_H_5_	0.00374	0.01070	0.00050
Type IV	C_6_H_5_Cl⋯ClC_6_H_5_	0.00603	0.01676	0.00052
	C_6_H_5_Br⋯BrC_6_H_5_	0.00516	0.01368	0.00065
	C_6_H_5_I⋯IC_6_H_5_	0.00439	0.00905	0.00036

**Table 4:** Electrostatic (*E*_elst_), induction (*E*_ind_), dispersion (*E*_disp_), exchange (*E*_exch_), total SAPT2+(3) binding energy (*E*_SAPT2+(3)_), and the energy difference (∆∆*E*) between MP2 and SAPT2+(3) energies for C_6_H_5_X⋯XC_6_H_5_ homodimers (where X = Cl, Br, and I) in the fashion of type I–IV halogen⋯halogen interactions at the most favorable parameters ^a^.

Homodimer		*E*_elst_	*E*_ind_	*E*_disp_	*E*_exch_	*E*_SAPT2+(3)_^b^	∆∆*E* ^c^
Type I	C_6_H_5_Cl⋯ClC_6_H_5_	–0.21	–0.17	–2.43	1.64	–1.17	0.41
	C_6_H_5_Br⋯BrC_6_H_5_	–0.74	–0.30	–2.12	1.91	–1.24	0.09
	C_6_H_5_I⋯IC_6_H_5_	–1.13	–0.53	–2.65	2.62	–1.68	0.19
Type II	C_6_H_5_Cl⋯ClC_6_H_5_	–0.08	–0.14	–2.19	1.26	–1.15	–0.17
	C_6_H_5_Br⋯BrC_6_H_5_	–0.67	–0.27	–2.03	1.74	–1.24	–0.56
	C_6_H_5_I⋯IC_6_H_5_	–1.61	–0.77	–3.59	3.60	–2.36	0.16
Type III	C_6_H_5_Cl⋯ClC_6_H_5_	0.09	–0.10	–1.44	0.77	–0.68	0.08
	C_6_H_5_Br⋯BrC_6_H_5_	0.17	–0.15	–1.75	0.91	–0.82	0.08
	C_6_H_5_I⋯IC_6_H_5_	0.32	–0.24	–1.99	0.98	–0.93	0.14
Type IV	C_6_H_5_Cl⋯ClC_6_H_5_	–0.28	–0.18	–2.67	1.83	–1.30	0.03
	C_6_H_5_Br⋯BrC_6_H_5_	–0.27	–0.18	–2.65	1.80	–1.30	0.05
	C_6_H_5_I⋯IC_6_H_5_	–0.33	–0.19	–2.54	1.77	–1.29	0.13

^a^ all energy terms are in kcal/mol; ^b^ $E_{\mathrm{SAPT}2+\left(3\right)}=E_{\mathrm{elst}}+E_{\mathrm{ind}}+E_{\mathrm{disp}}+E_{\mathrm{exch}}$; ^c^ $\Delta \Delta E=E_{\mathrm{MP}2/\mathrm{aug}-\mathrm{cc}-\mathrm{pVDZ}\left(\mathrm{PP}\right)}-E_{\mathrm{SAPT}2+\left(3\right)}$.

**Table 5:** Binding energies (in kcal/mol) for the *trans* (*E_trans_*) and *cis* (*E_cis_*) geometries in addition to the maximum negative binding energy (*E*_max_) for the *trans → cis* interconversion of C_6_H_5_X⋯XC_6_H_5_ homodimers (where X = Cl, Br, and I) in the pattern of type IV halogen⋯halogen interaction.

Homodimer	*trans* Configuration	*cis* Configuration	*Trans → cis* Interconversion	
	*E_trans_* (kcal/mol)	*E_cis_* (kcal/mol)	*E*_max_ (kcal/mol)	*Φ*^a^ (Degree)
C_6_H_5_Cl⋯ClC_6_H_5_	–1.27	–4.20	–4.91	25°
C_6_H_5_Br⋯BrC_6_H_5_	–1.25	–3.96	–4.25	20°
C_6_H_5_I⋯IC_6_H_5_	–1.16	–2.93	–2.93	10°

^a^ Torsional angle of C-X⋯X-C (see Figure 9 for details).

## Data Availability

Not applicable.

## Supplementary Materials

The following supporting information can be downloaded at: https://www.mdpi.com/article/10.3390/ijms23063114/s1.
