# Supplementary material for: Type I–IV Halogen⋯Halogen Interactions: A Comparative Theoretical Study in Halobenzene⋯Halobenzene Homodimers
Source: Int J Mol Sci. 2022 Mar 14;23(6):3114. doi: 10.3390/ijms23063114 (PMC8953242; doi:10.3390/ijms23063114)
Supplement: Supplementary file 1 [file ijms-23-03114-s001.zip › ijms-1635886-supplementary.pdf]

Type I–IV Halogen···Halogen Interactions: A Comparative Theoretical Study in Halobenzene···Halobenzene Homodimers

Mahmoud A. A. Ibrahim [1,*], Rehab R. A. Saeed [1], Mohammed N. I. Shehata [1], Muhammad Naeem Ahmed [2], Ahmed M. Shawky [3], Manal M. Khowdiary [4], Eslam B. Elkaeed [5], Mahmoud E. S. Soliman [6,*] and Nayra A. M. Moussa [1]

[1] Computational Chemistry Laboratory, Chemistry Department, Faculty of Science, Minia University, Minia 61519, Egypt; r.saeed@compchem.net (R.R.A.S.); m.shehata@compchem.net (M.N.I.S.); n.moussa@compchem.net (N.A.M.M.)
[2] Department of Chemistry, The University of Azad Jammu and Kashmir, Muzaffarabad 13100, Pakistan; drnaeem@ajku.edu.pk
[3] Science and Technology Unit (STU), Umm Al-Qura University, Makkah 21955, Saudi Arabia; amesmail@uqu.edu.sa
[4] Chemistry Department, Faculty of Applied Science, Umm Al-Qura University, Al-Lith Branch, Makkah 24211, Saudi Arabia; mmkhowdairy@uqu.edu.sa
[5] Department of Pharmaceutical Sciences, College of Pharmacy, AlMaarefa University, Riyadh 13713, Saudi Arabia; ikaeed@mcst.edu.sa
[6] Molecular Modelling and Drug Design Research Group, School of Health Sciences, University of KwaZulu-Natal, Westville, Durban 4000, South Africa
* Correspondence: m.ibrahim@compchem.net (M.A.A.I.); soliman@ukzn.ac.za (M.E.S.S.)

Table S1. Cartesian atomic coordinates of $C_6H_5X\cdots XC_6H_5$ homodimers (where X = Cl, Br, and I) in the fashion of type I–IV halogen···halogen interactions at the most favorable parameters.

Type I

$C_6H_5Cl\cdots ClC_6H_5$

C	-0.19872095	0.00020035	4.54919767
C	-0.35467905	1.20292771	3.83462238
C	-0.29464516	1.21157312	2.42882085
C	-0.07646219	0.00000000	1.75127232
C	0.08174204	-1.21142685	2.44540334
C	0.01896358	-1.20258415	3.85113764
H	-0.24643068	0.00032635	5.64155531
H	-0.52419603	2.14178658	4.36970139
H	-0.41439149	2.13951087	1.86423171
H	0.25029624	-2.13951087	1.89366102
H	0.14112428	-2.14143729	4.39897966
Cl	0.00000000	0.00000000	0.00000000
C	3.90859176	-0.00022092	-4.54920340
C	4.07868649	-1.20066369	-3.83401060
C	4.01875521	-1.20929241	-2.42820501
C	3.78646208	0.00000008	-1.75127220
C	3.61401893	1.20912516	-2.44602489
C	3.67684938	1.20029092	-3.85175705
H	3.95630933	-0.00034574	-5.64156055
H	4.25912453	-2.13775682	-4.36861324
H	4.14940859	-2.13546681	-1.86313939
H	3.43464376	2.13546014	-1.89475513
H	3.54362346	2.13735557	-4.40008307
Cl	3.71000005	0.00000003	0.00000003

$C_6H_5Br\cdots BrC_6H_5$

C	-2.17283988	-1.76255238	0.61501920
C	-1.60314703	-0.50653148	0.88685673
C	-2.19457340	0.38259536	1.80100679
C	-3.38150907	0.00000000	2.45346999
C	-3.96666217	-1.25333929	2.19431567
C	-3.35992098	-2.13007283	1.27580845
H	-1.69666851	-2.43875933	-0.09936072
H	-1.73512959	1.35445464	1.99781442
H	-3.84688950	0.68745738	3.16615462
H	-4.88889122	-1.54474950	2.70445991
H	-3.80846977	-3.10629535	1.06871259
Br	0.00000000	0.00000000	0.00000000
C	5.90454699	1.74565351	-0.58543128
C	5.31314732	0.50653148	-0.88685673
C	5.88286568	-0.36569569	-1.83059537
C	7.06994082	0.01679107	-2.48286843
C	7.67665077	1.25334823	-2.19433117
C	7.09146048	2.11330485	-1.24644923
H	5.44504906	2.40888023	0.15167432
H	5.40673901	-1.32456768	-2.05014181
H	7.51848723	-0.65756136	-3.21849775
H	8.59886623	1.54476929	-2.70449471
H	7.55684734	3.07641888	-1.01640320
Br	3.71000005	0.00000000	0.00000000

$C_6H_5I \cdots IC_6H_5$

C	-3.56104660	-2.36233568	0.99052978
C	-2.37379527	-1.89389288	0.39651117
C	-1.78202713	-0.71287328	0.88355231
C	-2.36067438	0.00000000	1.95089757
C	-3.54800534	-0.48141560	2.53426218
C	-4.15063906	-1.66032326	2.05795264
H	-4.02286148	-3.28007627	0.61368656
H	-1.91589463	-2.43923330	-0.43259549
H	-1.89261961	0.91570318	2.32089639
H	-3.99965739	0.07093211	3.36399364
H	-5.07291079	-2.02923083	2.51525497
I	0.00000000	0.00000000	0.00000000
C	7.56803467	2.37516117	-1.00642598
C	6.38069822	1.90680194	-0.41251069
C	5.80202725	0.71287328	-0.88355231
C	6.39373495	-0.01287286	-1.93494248
C	7.58097269	0.46863440	-2.51842117
C	8.17060425	1.66035879	-2.05799651
H	8.01968147	3.30292296	-0.64200306
H	5.91272022	2.46207523	0.40428486
H	5.93578198	-0.93853283	-2.29260015
H	8.04276325	-0.09370591	-3.33576727
H	9.09287598	2.02926540	-2.51529789
I	4.02000000	-0.00000001	0.00000001

<u>Type II</u>

$C_6H_5Cl\cdots ClC_6H_5$

C	0.00006478	2.01627183	4.08280993
C	-0.01097347	0.61186868	3.98781347
C	-0.01105339	-0.01908007	2.73009348
C	0.00000001	0.77626282	1.57169247
C	0.01111831	2.17947102	1.64438951
C	0.01106351	2.79457140	2.90999579
H	0.00006044	2.50035286	5.06321144
H	-0.01953226	0.00000000	4.89448071
H	-0.01956982	-1.10850024	2.64488792
H	0.01959171	2.77420783	0.72766352
H	0.01969530	3.88643765	2.97521567
Cl	-0.00000000	0.00000000	0.00000000
C	8.04353604	-0.00022021	0.00003655
C	7.33644375	-1.08655894	0.54901969
C	5.92936167	-1.09436738	0.55296636
C	5.24294075	-0.00000001	0.00000001
C	5.92951044	1.09419632	-0.55295420
C	7.33664498	1.08619368	-0.54893959
H	9.13693413	-0.00033218	0.00009670
H	7.87840781	-1.93459809	0.97751284
H	5.37053284	-1.93250811	0.97652221
H	5.37094054	1.93249536	-0.97654033
H	7.87863764	1.93418455	-0.97749037
Cl	3.49000010	0.00000000	-0.00000000

$C_6H_5Br\cdots BrC_6H_5$

C	0.02020041	-2.72640800	0.87884223
C	0.00000000	-1.38576150	1.30108964
C	-0.02020101	-1.04880798	2.66566157
C	-0.02007111	-2.08076358	3.62272549
C	-0.00001064	-3.42880940	3.21924758
C	0.02004422	-3.74657011	1.84845281
H	0.03571589	-2.96644211	-0.18721434
H	-0.03572538	0.00000000	2.97240782
H	-0.03573610	-1.82522082	4.68646812
H	-0.00002373	-4.22600222	3.96768403
H	0.03571309	-4.79211330	1.52644515
Br	-0.00000000	0.00000000	0.00000000
C	6.22918133	0.82409519	0.90723735
C	5.54083351	0.00000000	-0.00000000
C	6.22921233	-0.82407379	-0.90721524
C	7.63663153	-0.81826127	-0.90083462
C	8.34322171	0.00003641	0.00002312
C	7.63659100	0.81832987	0.90086865
H	5.67447476	1.45721459	1.60422611
H	5.67456440	-1.45721018	-1.60423505
H	8.17844872	-1.45738316	-1.60443532
H	9.43669037	0.00006939	0.00003908
H	8.17841248	1.45744669	1.60446918
Br	3.63999991	0.00000000	-0.00000000

$C_6H_5I\cdots IC_6H_5$

C	-0.00277313	2.04334044	3.88169885
C	-0.00278729	1.06175518	2.87256384
C	-0.00000000	1.46415758	1.52339005
C	0.00275310	2.82821941	1.17471433
C	0.00273053	3.79769373	2.19547343
C	-0.00003389	3.41030169	3.54816794
H	-0.00493747	1.73299885	4.93108606
H	-0.00491310	0.00000000	3.13171291
H	0.00490175	3.12916446	0.12404102
H	0.00486772	4.85855484	1.92695785
H	-0.00003327	4.16809464	4.33655405
I	-0.00000000	0.00000000	-0.00000000
C	8.17458016	-0.87523597	0.84505129
C	6.76681906	-0.88130164	0.85090077
C	6.07293040	-0.00000001	0.00000000
C	6.76676899	0.88135791	-0.85090744
C	8.17451817	0.87534589	-0.84509790
C	8.88134629	0.00007223	-0.00002243
H	8.71612126	-1.55933893	1.50555682
H	6.21791655	-1.56122136	1.50733232
H	6.21779019	1.56122959	-1.50732446
H	8.71604782	1.55947268	-1.50558865
H	9.97487408	0.00010329	-0.00005314
I	3.96000010	0.00000000	-0.00000000

Type III

$C_6H_5Cl\cdots ClC_6H_5$

C	-4.55353594	0.00006667	0.00021302
C	-3.84644341	-0.00000322	1.21738780
C	-2.43936157	-0.00000439	1.22613692
C	-1.75294065	0.00000000	0.00000000
C	-2.43951035	0.00007073	-1.22597873
C	-3.84664488	0.00009661	-1.21702540
H	-5.64693546	0.00006348	0.00034009
H	-4.38840771	0.00000000	2.16753364
H	-1.88053286	-0.00005841	2.16522121
H	-1.88094079	0.00008032	-2.16521811
H	-4.38863754	0.00016648	-2.16715431
Cl	0.00000000	0.00000000	0.00000000
C	8.13353642	0.00006665	-0.00021307
C	7.42644318	0.00007112	-1.21738791
C	6.01936158	0.00007068	-1.22613704
C	5.33294066	0.00000003	0.00000000
C	6.01951035	-0.00000443	1.22597873
C	7.42664536	0.00002207	1.21702564
H	9.22693547	0.00006347	-0.00034014
H	7.96840771	0.00013276	-2.16753340
H	5.46053275	0.00007392	-2.16522121
H	5.46094092	-0.00005225	2.16521811
H	7.96863755	0.00003372	2.16715407
Cl	3.58000001	-0.00000004	-0.00000004

$C_6H_5Br\cdots BrC_6H_5$

C	-2.58918166	0.00000330	-1.22564769
C	-1.90083396	0.00000000	-0.00000000
C	-2.58921218	-0.00000222	1.22561693
C	-3.99663162	0.00001019	1.21698582
C	-4.70322132	0.00001133	-0.00004156
C	-3.99659085	0.00001794	-1.21705699
H	-2.03447485	0.00000957	-2.16725993
H	-2.03456450	0.00000000	2.16726351
H	-4.53844881	0.00000622	2.16752839
H	-5.79668951	0.00002504	-0.00007578
H	-4.53841209	0.00001789	-2.16759610
Br	-0.00000000	-0.00000000	0.00000000
C	6.35918123	0.00000337	-1.22564781
C	5.67083342	-0.00000004	0.00000005
C	6.35921223	-0.00000454	1.22561705
C	7.76663239	-0.00001682	1.21698594
C	8.47322113	-0.00001134	-0.00004156
C	7.76659042	-0.00001128	-1.21705699
H	5.80447514	0.00000235	-2.16725993
H	5.80456430	-0.00001172	2.16726375
H	8.30844910	-0.00001802	2.16752815
H	9.56668884	-0.00002504	-0.00007567
H	8.30841190	-0.00000608	-2.16759586
Br	3.77000005	-0.00000000	0.00000001

$C_6H_5I\cdots IC_6H_5$

C	-4.21457958	-0.00001876	1.21661401
C	-2.80681872	-0.00001384	1.22504067
C	-2.11293030	-0.00000000	0.00000000
C	-2.80676866	-0.00002048	-1.22508585
C	-4.21451855	-0.00002408	-1.21672547
C	-4.92134666	-0.00003404	-0.00006755
H	-4.75612020	-0.00003025	2.16754222
H	-2.25791645	0.00000000	2.17012978
H	-2.25778985	-0.00001134	-2.17013025
H	-4.75604820	-0.00003976	-2.16766071
H	-6.01487446	-0.00003352	-0.00011123
I	-0.00000000	0.00000000	0.00000000
C	8.36457996	-0.00002533	-1.21661401
C	6.95681839	-0.00002046	-1.22504067
C	6.26293020	-0.00000000	0.00000000
C	6.95676832	-0.00001381	1.22508585
C	8.36451893	-0.00001752	1.21672547
C	9.07134609	-0.00003405	0.00006755
H	8.90612106	-0.00004196	-2.16754222
H	6.40791636	-0.00001174	-2.17012978
H	6.40778952	0.00000037	2.17013025
H	8.90604763	-0.00002807	2.16766071
H	10.16487484	-0.00003352	0.00011122
I	4.14999991	-0.00000000	0.00000000

Type IV

$C_6H_5Cl \cdots ClC_6H_5$

C	-0.00005985	0.00021482	4.55353594
C	0.03903949	1.21676171	3.84644341
C	0.03932110	1.22550631	2.43936157
C	0.00000000	0.00000000	1.75294065
C	-0.03938233	-1.22534597	2.43951011
C	-0.03912111	-1.21639657	3.84664512
H	-0.00005258	0.00034189	5.64693546
H	0.06950318	2.16641879	4.38840771
H	0.06948742	2.16410589	1.88053286
H	-0.06950921	-2.16410184	1.88094079
H	-0.06965741	-2.16603470	4.38863754
Cl	-0.00000000	0.00000000	-0.00000000
C	3.67994007	-0.00021505	-4.55353594
C	3.71903952	-1.21676159	-3.84644365
C	3.71932133	-1.22550631	-2.43936157
C	3.67999991	0.00000000	-1.75294065
C	3.64061769	1.22534597	-2.43951011
C	3.64087900	1.21639657	-3.84664512
H	3.67994722	-0.00034189	-5.64693546
H	3.74950298	-2.16641879	-4.38840771
H	3.74948772	-2.16410589	-1.88053286
H	3.61049088	2.16410208	-1.88094091
H	3.61034282	2.16603470	-4.38863754
Cl	3.67999991	-0.00000000	-0.00000000

$C_6H_5Br\cdots BrC_6H_5$

C	0.02020041	-0.00000000	2.86455321
C	-0.00000000	0.81319344	1.71810520
C	-0.02020101	2.21533489	1.81605089
C	-0.02007111	2.80964065	3.09186602
C	-0.00001064	2.01204157	4.25111485
C	0.02004422	0.60986573	4.13299274
H	0.03571589	-1.08828735	2.76594615
H	-0.03572538	2.82906127	0.91193187
H	-0.03573610	3.90048385	3.17500234
H	-0.00002373	2.47980618	5.23948240
H	0.03571309	-0.01738455	5.02932215
Br	-0.00000000	0.00000000	0.00000000
C	3.99020059	0.00000021	-2.86455321
C	3.97000010	-0.81319344	-1.71810520
C	3.94979913	-2.21533489	-1.81605089
C	3.94992907	-2.80964065	-3.09186602
C	3.96998937	-2.01204157	-4.25111485
C	3.99004442	-0.60986555	-4.13299274
H	4.00571615	1.08828735	-2.76594615
H	3.93427474	-2.82906127	-0.91193187
H	3.93426377	-3.90048385	-3.17500234
H	3.96997625	-2.47980618	-5.23948240
H	4.00571329	0.01738455	-5.02932215
Br	3.97000010	0.00000000	0.00000000

$C_6H_5I\cdots IC_6H_5$

C	-0.01210884	0.00000000	4.38664818
C	-0.01218765	-0.39851150	3.03644466
C	-0.00000000	0.58598119	2.03004932
C	0.01215381	1.95537734	2.35693407
C	0.01206709	2.33775806	3.71178246
C	-0.00003338	1.36490738	4.72828531
H	-0.02157014	-0.76339620	5.17065573
H	-0.02156562	-1.45871174	2.77116299
H	0.02155427	2.71105814	1.56741202
H	0.02150129	3.40153027	3.96835947
H	-0.00003242	1.66821861	5.77890682
I	-0.00000000	0.00000000	-0.00000000
C	4.41789129	-0.00000013	-4.38664865
C	4.41781262	0.39851150	-3.03644466
C	4.43000010	-0.58598113	-2.03004909
C	4.44215420	-1.95537734	-2.35693407
C	4.44206694	-2.33775806	-3.71178246
C	4.42996672	-1.36490726	-4.72828484
H	4.40843037	0.76339608	-5.17065620
H	4.40843466	1.45871174	-2.77116299
H	4.45155457	-2.71105814	-1.56741202
H	4.45150164	-3.40153027	-3.96835947
H	4.42996767	-1.66821861	-5.77890682
I	4.43000010	0.00000000	-0.00000000
